# Yaws

**Published:** 1910-01-15

**Authors:** 


### Tropical Medicine.

### YAWS.

Yaws is an interesting disease that has an extensive geographical distribution in many parts of the tropics. It is especially common in many of the islands in the West Indies and the West Coast of Africa. It occurs in Ceylon and in many of the Pacific Islands, notably Fiji, and it is prevalent in Assam and Burmah. It is also known under the names of Frambœsia, Paranga (Ceylon), Puru (Malay), Pian (Indo-China), Coko (Fiji). Little is known at present of its distribution in India; its existence there has been confirmed, but its exact extent requires investigation.

It is due to the efforts of Castellani that light has been thrown upon the ætiology of yaws; for it was in February 1905 that this observer announced his discovery of a delicate spirochæte in the secretion obtained from an ulcer in a case of yaws. In a paper read before the Ceylon Branch of the British Medical Association, he named this organism the spirochæta pertenuis. Castellani's observation was confirmed by Wellman, Powell, and others. Castellani has also found spirochætes which show differences in morphological details in the ulcerative lesions of yaws. These spirochætes are of a coarser variety and are termed S. obtusa and S. acumina. By using Leishman's method and allowing the alcoholic solution to act on the film for five or ten minutes, and the subsequent admixture with distilled water to act for half-an-hour to several hours, good staining effects can be obtained. Giemsa's stain also gives good results. In some preparations obtained by Lœffler's method of staining for flagella, Castellani observed in some parasites the presence of an extremely delicate flagellum at one end, and he considers, therefore, that the organism should be considered as a treponema instead of a spirochæte.

That the treponema pertenue is the causative factor of this disease is now well established. Neisser, Baermann, and Halberstädter showed that monkeys can be successfully inoculated with material obtained from the granulomata of a typical case of yaws, and from the glands of the arm of a case in full eruption. In the lower monkeys the first symptoms appeared in 22 to 91 days after inoculation, and in the anthropoid apes the symptoms appeared in 13 days. Once in three attempts the authors succeeded in infecting another monkey from the first. These observers further noted an important point, namely, that a monkey can be infected with yaws fifteen days after the appearance of a syphilitic chancre, proving that monkeys inoculated with syphilis are not immune to yaws. Castellani, in some further observations, showed that the infection of the yaws in monkeys is a general one, spirochætes being found in the spleen and lymph glands. He confirmed the observation of Neisser and others that monkeys successfully inoculated with syphilis do not become immune to yaws, and *vice versa*; and by means of the Bordet-Gengou reaction he detected specific yaws antibodies and antigen. Further, he proved that the specific yaws antibodies and antigen are entirely different from syphilitic antibodies and antigen, thus adding to the chain of evidence that yaws and syphilis are two entirely different entities. Ashburn and Craig further support the work of Castellani, and undoubtedly their experiments prove that the treponema pertenue is constantly present in the lesions of the yaws.

Yaws not only differs from syphilis in its clinical features, but also in its geographical distribution. The most important points bearing on the specific entity of these two diseases may be briefly summarised:—(1) In non-ulcerative papules, in the spleen, in the lymphatic glands of yaws, in patients as well as in inoculated monkeys, the treponema pertenue is the only organism present. (2) The extract of yaws material containing the treponema pertenue is infective to monkeys. (3) The extract of yaws from which the treponema pertenue has been removed by filtration becomes inert, and monkeys inoculated with it do not contract the disease. Neisser, while pointing out the directions in which yaws and syphilis resemble one another, decides that yaws cannot be merely a degenerative form of syphilis, because neither protects against the other. The communicability of yaws still requires investigation. It is well known that in most cases it is conveyed by direct contact from person to person, usually by absorption of the virus through a pre-existing abrasion or wound on the surface of the skin. Among the natives in Ceylon the primary lesion frequently develops in women on the skin of the trunk just above the hip, due, no doubt, to their method of carrying their children astride of their hips. According to Jeanselme, the disease is propagated among the adult natives in Indo-China by the chopsticks used instead of forks, the water-pipe which is handed from mouth to mouth, and the sleeping-mats common to all. The rôle of biting-flies in spreading this disease still requires to be investigated. Castellani has shown that under certain conditions the disease may be spread by house flies. Modder suggests that ticks are concerned in its spread.

As regards treatment, Castellani has found, in obstinate cases, the mixed method of treatment to be the best. That observer obtained the most satisfactory results with iodide of potassium, followed either by atoxyl or quinine or sodium cacodylate. The iodide of potassium was administered in doses of 1 gramme in milk thrice daily; the atoxyl by daily subcutaneous injections of 0.05 gramme, and one injection of cacodylate of soda or quinine cacodylate containing 0.05 gramme of the drug. As regards local treatment the same observer recommends the application of perchloride of mercury 1 in 1,000, and dusting the granulomata with iodoform or boric acid. For ulcerative lesions he recommends the use of 20 per cent. protargol ointment. Campbell Graham obtained encouraging results by using sodium bicarbonate internally in 4-gram doses, together with the local application of copper sulphate.

